# Supplementary material for: Climate-induced decline in the quality and quantity of European hops calls for immediate adaptation measures
Source: Nat Commun. 2023 Oct 10;14:6028. doi: 10.1038/s41467-023-41474-5 (PMC10564787; doi:10.1038/s41467-023-41474-5)
Supplement: Supplementary file 1 — Supplementary information [file 41467_2023_41474_MOESM1_ESM.pdf]

Supplemental information

Supplementary Table 1

Supplementary Tab. 1 Description of the model used for modelling yields and alpha content of hops

Hop parameter	Formula
Alpha content in % of mean value<br><br>T – difference in seasonal daily temperatures<br><br>Tmin = -10 °C<br><br>Tmax = 15 °C	$Alpha\ content = 0.01831 * T^3 - 0.3881 * T^2$- 1.1594 * T + 114.11<br><br>Alpha content [%]
Yield in % of mean value<br><br>R – difference in seasonal mean rainfall<br><br>Rmin = -45%<br><br>Rmax = 90%	$Yield = -0.001 * R^3 + 0.0014 * R^2 + 0.7274 * R + 102.51$<br><br>Yield [%]

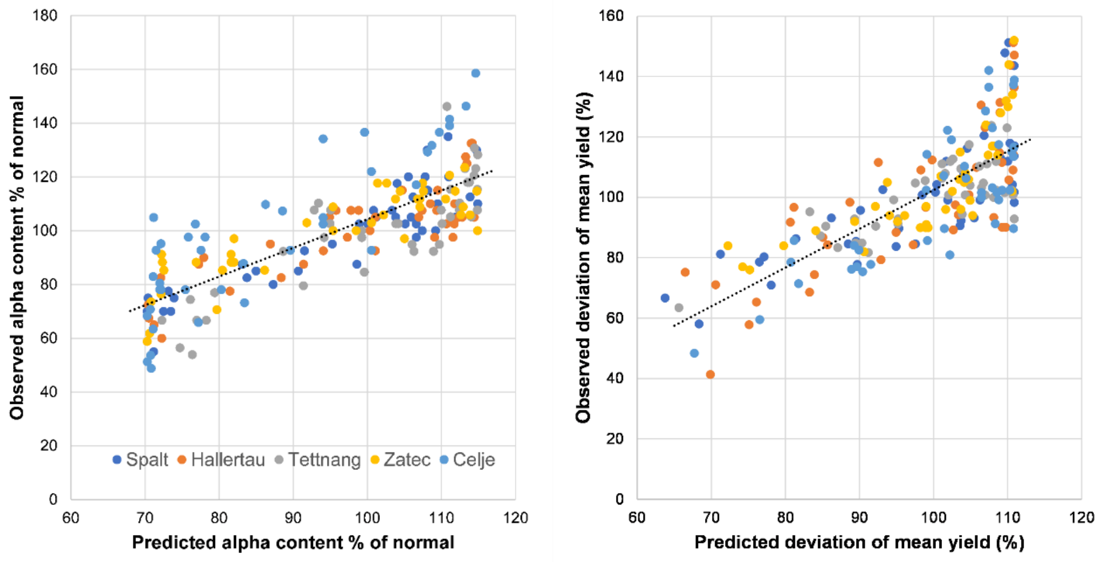

Supplementary Fig. 1. Predicted vs. observed regression point plots of alpha level (left) and hop yield (right). Each graph shows the predicted value vs. the actual value for each observation at all selected locations. Spalt (blue dot), Hallertau (orange dot), Tettnang (grey dot), Zatec (yellow dot) and Celje (sky blue dot).

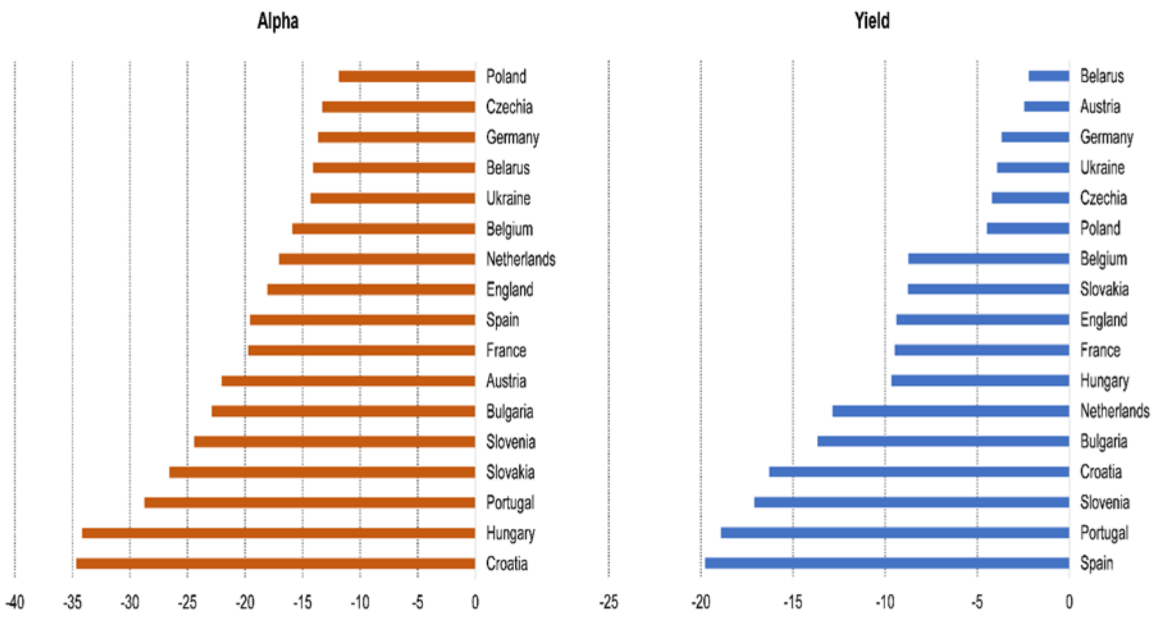

Supplementary Fig. 2. Estimated reduction in alpha content and yield at the country level as the

% of the baseline (1989-2018). Median calculated for the period 2021-2050: yields (fill blue), alpha (fill orange).
