# Supplementary material for: Climate-induced decline in the quality and quantity of European hops calls for immediate adaptation measures
Source: Nat Commun. 2023 Oct 10;14:6028. doi: 10.1038/s41467-023-41474-5 (PMC10564787; doi:10.1038/s41467-023-41474-5)
Supplement: Supplementary file 2 — Reporting Summary [file 41467_2023_41474_MOESM2_ESM.pdf]

nature portfolio

Corresponding author(s): Martin Mozny
Last updated by author(s): Sep 3, 2023

Reporting Summary

Nature Portfolio wishes to improve the reproducibility of the work that we publish. This form provides structure for consistency and transparency in reporting. For further information on Nature Portfolio policies, see our Editorial Policies and the Editorial Policy Checklist.

Statistics

For all statistical analyses, confirm that the following items are present in the figure legend, table legend, main text, or Methods section.

n/a	Confirmed	
[ ]	[x]	The exact sample size (*n*) for each experimental group/condition, given as a discrete number and unit of measurement
[x]	[ ]	A statement on whether measurements were taken from distinct samples or whether the same sample was measured repeatedly
[x]	[ ]	The statistical test(s) used AND whether they are one- or two-sided<br>*Only common tests should be described solely by name; describe more complex techniques in the Methods section.*
[x]	[ ]	A description of all covariates tested
[x]	[ ]	A description of any assumptions or corrections, such as tests of normality and adjustment for multiple comparisons
[ ]	[x]	A full description of the statistical parameters including central tendency (e.g. means) or other basic estimates (e.g. regression coefficient) AND variation (e.g. standard deviation) or associated estimates of uncertainty (e.g. confidence intervals)
[ ]	[x]	For null hypothesis testing, the test statistic (e.g. *F*, *t*, *r*) with confidence intervals, effect sizes, degrees of freedom and *P* value noted<br>*Give P values as exact values whenever suitable.*
[x]	[ ]	For Bayesian analysis, information on the choice of priors and Markov chain Monte Carlo settings
[x]	[ ]	For hierarchical and complex designs, identification of the appropriate level for tests and full reporting of outcomes
[x]	[ ]	Estimates of effect sizes (e.g. Cohen's *d*, Pearson's *r*), indicating how they were calculated

Our web collection on statistics for biologists contains articles on many of the points above.

Software and code

Policy information about availability of computer code

Data collection	Curve fitting as described in the text with the toolbox in R
Data analysis	R, Statistica, ESRI GIS, MS Excel (all - freely available libraries, toolbox, no custom software in the code)

For manuscripts utilizing custom algorithms or software that are central to the research but not yet described in published literature, software must be made available to editors and reviewers. We strongly encourage code deposition in a community repository (e.g. GitHub). See the Nature Portfolio guidelines for submitting code & software for further information.

Data

Policy information about availability of data

All manuscripts must include a data availability statement. This statement should provide the following information, where applicable:

- Accession codes, unique identifiers, or web links for publicly available datasets
- A description of any restrictions on data availability
- For clinical datasets or third party data, please ensure that the statement adheres to our policy

All data that support the findings of this study are publicly available in FigShare (https://doi.org/ 10.6084/m9.figshare.23180342)

Research involving human participants, their data, or biological material

Policy information about studies with human participants or human data. See also policy information about sex, gender (identity/presentation), and sexual orientation and race, ethnicity and racism.

Reporting on sex and gender	Research does not involving animals or humans
Reporting on race, ethnicity, or other socially relevant groupings	not applicable for this study
Population characteristics	not applicable for this study
Recruitment	not applicable for this study
Ethics oversight	not applicable for this study

Note that full information on the approval of the study protocol must also be provided in the manuscript.

Field-specific reporting

Please select the one below that is the best fit for your research. If you are not sure, read the appropriate sections before making your selection.

☐ Life sciences ☐ Behavioural & social sciences ☒ Ecological, evolutionary & environmental sciences

For a reference copy of the document with all sections, see nature.com/documents/nr-reporting-summary-flat.pdf

Ecological, evolutionary & environmental sciences study design

All studies must disclose on these points even when the disclosure is negative.

Study description	Evaluation and prediction of a relationship between weather conditions and hop yields/quality
Research sample	Existing dataset - hop acreages, average yields and alpha content (the Barth-Haas reports), daily precipitation totals, average daily temperatures, and onset of hop cone development (the German, Czech and Slovenian national meteorological services). The data and their sources are described in the Methodology in the submitted article.
Sampling strategy	We used all of the available hop yield/quality data from 1971
Data collection	Hop acreages, average yields and alpha content (bitter substances in aromatic hops) for the individual areas were obtained from the Barth-Haas reports for 1970–2018 (yearly data). Daily precipitation totals, average daily temperatures (daily data 1.1.1970 -31.12.2018), and onset of hop cone development (yearly data 1970-2018) were obtained from the German, Czech and Slovenian national meteorological services. All data without gaps, full.
Timing and spatial scale	1970–2018, average hop yield/quality values for the hop-farming regions of Hallertau (DE), Spalt (DE), Tettnang (DE), Zatec/Saaz (CZ) and Celje (SLO)
Data exclusions	No data were excluded from the analyses
Reproducibility	All attempts to repeat the experiments were successful according to available data and algorithms used in the study
Randomization	Not applicable - we used all data for selected study areas
Blinding	Data blinding was not performed

Did the study involve field work? ☐ Yes ☒ No

Reporting for specific materials, systems and methods

We require information from authors about some types of materials, experimental systems and methods used in many studies. Here, indicate whether each material, system or method listed is relevant to your study. If you are not sure if a list item applies to your research, read the appropriate section before selecting a response.

Materials & experimental systems

n/a	Involved in the study
☒	☐ Antibodies
☒	☐ Eukaryotic cell lines
☒	☐ Palaeontology and archaeology
☒	☐ Animals and other organisms
☒	☐ Clinical data
☒	☐ Dual use research of concern
☒	☐ Plants

Methods

n/a	Involved in the study
☒	☐ ChIP-seq
☒	☐ Flow cytometry
☒	☐ MRI-based neuroimaging
